# ﻿Using scanning electron microscopy and molecular data to discover a new species from old herbarium collections: The case of *Phlomoideshenryi* (Lamiaceae, Lamioideae)

**DOI:** 10.3897/phytokeys.238.117180

**Published:** 2024-02-20

**Authors:** Yue Zhao, Fei Zhao, Alan J. Paton, Jin-Fei Xiao, Ya-Ping Chen, Chun-Lei Xiang

**Affiliations:** 1 Ministry of Education Key Laboratory of Ecology and Resource Use of the Mongolian Plateau & Inner Mongolia Key Laboratory of Grassland Ecology, School of Ecology and Environment, Inner Mongolia University, Hohhot 010000, Inner Mongolia, China Inner Mongolia University Hohhot China; 2 CAS Key Laboratory for Plant Diversity and Biogeography of East Asia, Kunming Institute of Botany, Chinese Academy of Sciences, Kunming, 650201, China Kunming Institute of Botany, Chinese Academy of Sciences Kunming China; 3 CAS Key Laboratory of Mountain Ecological Restoration and Bioresource Utilization & Ecological Restoration and Biodiversity Conservation Key Laboratory of Sichuan Province, Chinese Academy of Sciences, Chengdu Institute of Biology, Chengdu, Sichuan, China Chinese Academy of Sciences, Chengdu Institute of Biology Chengdu China; 4 Royal Botanic Gardens, Kew, Richmond, TW9 3AB, UK Royal Botanic Gardens Kew United Kingdom; 5 University of Chinese Academy of Sciences, Beijing, China University of Chinese Academy of Sciences Beijing China

**Keywords:** Micromorphology, *Phlomoides*, *Phlomis*, Phlomideae, taxonomy, trichomes

## Abstract

*Phlomoides* is one of the largest genera of Lamiaceae with approximately 150–170 species distributed mainly in Eurasia. In this study, we describe and illustrate a new species, *P.henryi*, which was previously misidentified as *P.bracteosa*, from Yunnan Province, southwest China. Molecular phylogenetic analyses revealed that *P.henryi* is found within a clade in which most species lack basal leaves. In this clade, the new species is morphologically distinct from *P.rotata* in having an obvious stem and, from the rest, by having transparent to white trichomes inside the upper corolla lip. In addition, micro-features of trichomes on the calyx and leaf epidermis can differentiate the new species from other species grouped in the same clade and a key, based on trichome morphology for these species, is provided. The findings demonstrate that the use of scanning electron microscopy can reveal inconspicuous morphological affinities amongst morphologically similar species and play an important role in the taxonomic study of the genus *Phlomoides*.

## ﻿Introduction

The tribe Phlomideae (Lamiaceae, Lamioideae) was originally established by Scheen et al. (2010) to include seven genera, *Eremostachys* Bunge, *Lamiophlomis* Kudô, *Notochaete* Benth., *Phlomis* L., *Phlomoides* Moench, *Paraeremostachys* Adylov et al. and *Pseuderemostachys* Popov. Subsequent phylogenetic and taxonomic studies (Bendiksby et al. 2011; Mathiesen et al. 2011; Salmaki et al. 2012b) have revised generic boundaries and *Phlomoides* was expanded to include *Eremostachys*, *Lamiophlomis*, *Notochaete*, *Paraeremostachys* and *Pseuderemostachys*. Recently, two monotypic genera, *Metastachydium* Airy Shaw ex C.Y. Wu & H.W. Li and *Pseudomarrubium* Popov were also transferred to *Phlomoides* (Zhao et al. 2023a, b), leaving only two genera retained in Phlomideae, *Phlomoides* and *Phlomis*. The re-defined *Phlomoides* is easily distinguished from its sister genus *Phlomis* by: (1) being generally perennial herbs (vs. small shrubs, occasionally perennial herbs); (2) having leaves cordate to triangular-ovate, simple or laciniate to bipinnatisect (vs. lanceolate to oblong-lanceolate, not deeply lobed); (3) the upper lip of corolla dome-shaped with apex hairy or fringed-incised (vs. laterally compressed, flattened, sickle-shaped, apex not fringed-incised) and (4) a basic chromosome number of x = 11 (vs. x = 10) (Azizian and Cutler 1982; Astanova 1984; Ghaffari 2006; Fang et al. 2007; Mathiesen et al. 2011; Salmaki et al. 2012a; Zhao et al. 2021b).

As currently defined, *Phlomoides* consists of approximately 150–170 species and ranks the second largest genus within subfamily Lamioideae (Salmaki et al. 2012a, b; F Zhao et al. 2021). Species of *Phlomoides* are mainly distributed from central Europe to the Russian Far East, but highly diversified in three regions: Central Asia (59 spp.; Czerepanov (1995)), the Iranian highlands (ca. 41 spp.; Salmaki et al. (2012a)) and China (58 spp.; Xiang et al. (2014); Zhao et al. (2021a, 2024)). In China, most species are found in the southwest region and 29 species and 11 varieties are endemic and geographically restricted (Li and Hedge 1994). The existing infrageneric classification of Chinese *Phlomoides* (= PhlomissectionPhlomoides Briq.) was established by Hsuan (1977), who divided Chinese species into two subsections and 17 series, based on external morphology (e.g. the absence/presence of the basal leaves, shape of stem leaves, length and density of trichomes on stems and leaves etc.). However, most infrageneric categories were not recovered as monophyletic (Zhao et al. 2024) and those external and quantitative characters used for traditional taxonomy are highly variable amongst different species or at different populations for the same species. In contrast, some micro-features probably have taxonomic significance within *Phlomoides*. For example, Seyedi and Salmaki (2015) and Khosroshahi and Salmaki (2019) found trichome morphology to be important for the delimitation of sections and species of *Phlomoides*. In addition, trichome characters have significant taxonomic values in other genera of Lamiaceae (Gairola et al. 2009; Xiang et al. 2010; Hu et al. 2012; Yao et al. 2013). However, micro-features of trichomes and other characters of Chinese *Phlomoides* species are poorly known.

During the past ten years, phylogenetic and taxonomic studies have focused on *Phlomoides* from China (Xiang et al. 2014; Zhao et al. 2021a, b, 2023a, b, 2024) resolving some taxonomic puzzles. In the process of the continuing taxonomic study of the genus, two collections attracted our attention when investigating historical specimens. One collection with three sheets were collected by Augustine Henry in 1898 (*A. Henry 10216*) from Mengtze (now Mengzi County), Yunnan Province and were identified as *P.bracteosa* (Royle ex Benth.) Kamelin & Makhm. (= *Phlomisbracteosa* Royle ex Benth.). Two sheets were deposited at K (*A. Henry 10216A*, K000928267; *A. Henry 10216* K, without barcode) and another at LE (without barcode). Another collection was collected by F. Ducloux in 11 August 1907 (*F. Ducloux 369*) from Yunnan “Lao Kouy [Chan]” deposited at E (without barcode) without any identification, but external morphology indicates that this specimen represents the same species as the Henry’s collections at K and LE. However, characters (e.g. floral leaves with petioles 5–15 mm long, bracts subulate, simple long trichomes on calyces, bracts and both sides of leaves) shown in these specimens are obviously different from those of *P.bracteosa* (upper floral leaves sessile, lower floral leaves with petioles up to 20 mm long, bracts lanceolate-linear, branched trichomes on calyces, bracts and both sides of leaves). Fortunately, we re-discovered the plant in the wild from the possible locality where specimens were collected by Henry, after more than 125 years since the first collection in 1887. Molecular phylogenetic analyses and macro- and micro- morphological studies demonstrate that the species is a new species, *P.henryi* and we describe and illustrate it in this study.

## ﻿Materials and methods

### ﻿Taxon sampling

In total, we sampled 49 out of 58 (84.48%) Chinese species of *Phlomoides* for molecular phylogenetic analyses. Sampling is primarily based on previous molecular phylogenetic studies of *Phlomoides* (Zhao et al. 2024) and only samples of the potential new species and *P.bracteosa* were newly sequenced. Fresh leaves of the putative new species (*P.henryi*) were collected and dried with silica-gel in the field (Jianshui County, Yunnan Province) and herbarium materials of *P.bracteosa* were collected from the herbarium BM.

In addition, six species from the subclade comprising the potential new species, as well as *P.bracteosa*, were sampled to investigate macro-micro-features of trichomes on flora bracts and leaves. The list of sampled species and their origins are given in Table 1 and voucher specimens were deposited in the Herbarium of the Kunming Institute of Botany (KUN) and Institute of Botany (PE), Chinese Academy of Sciences.

**Table 1.** List of sampled *Phlomoides* species to investigate macro/micro features of trichomes and their voucher information.

Taxon	Geographical origin	Voucher information	Collection date
*P.henryi*	China, Yunnan Province, Honghe Hani and Yi Autonomous Prefecture, Jianshui County, on the forest edge, 23°57′52.54″N, 102°59′49.47″E, alt. 1279 m.	*F. Zhao et al. XCL2222* (KUN)	3 Sep. 2022
*P.bracteosa*	India, Uttarakhand, Bhyundar Valley, alt. 3430 m.	*Anonymous 6583* (PE)	16 Aug. 1975
*P.breviflora*	China, Tibet, Nielamu County, Qu Town, 28°4′44″N, 86°0′2.109″E, alt. 3246 m	*Y.P. Chen et al. EM1139* (KUN)	12 Sep. 2019
*P.macrophylla*	China, Xizang Province, Yadong County, on the way from Yadong to Nathu La Pass.	*Y.P. Chen et al. EM1094* (KUN)	9 Sep. 2019
*P.nyalamensis*	China, Xizang Province, Nyalam County, Zhangmu Town, on the way from Lixin to Xuebugang, 27°56′37.0356″N, 85°58′28.1712″E, alt. 2896 m.	*Y.P. Chen et al. EM1145* (KUN)	13 Sep. 2019
*P.tibetica*	China, Xizang Province, Linzhi City, Shergyla Mountain.	*C.L. Xiang et al. XCL1458* (KUN)	15 Sep. 2016
*P.milingensis*	China, Xizang Province, Linzhi City, Miling County, Lilong Town, Lilonggou, 29°1′45.6″N, 93°53′34.7″E, alt. 3188 m.	*C.L. Xiang et al. XCL1469* (KUN)	16 Sep. 2016
*P.rotata*	China, Xizang Province, Changdu City, Zuogong County, Dondara Mountain, 29°42′59.9″N, 98°1′7.3″E, alt. 5034 m.	*C.L. Xiang et al. XCL1419* (KUN)	12 Sep. 2016

### ﻿DNA extraction, selection of markers and molecular phylogenetic analyses

Total genomic DNA was extracted using the CTAB method (Doyle and Doyle 1987). Previous studies revealed that plastid DNA phylogeny can better resolve relationships of *Phlomoides* than the tree inferred from the nuclear ribosomal internal and external transcribed spacer regions (nrITS and nrETS) (Zhao et al. 2023a, b; 2024). In order to test systematic placement of the new species, nine plastid DNA regions (*atpB-rbcL*, *psbA-trnH*, *rpl16*, *rpl32-trnL*, *rps16*, *trnK*, *trnL-trnF*, *trnS-trnG*, *trnT-L*) were selected for phylogenetic reconstruction. Primers, polymerase chain reaction (PCR), sequencing and alignment followed those described in Zhao et al. (2024). The sequences newly generated in this study together with their GenBank accession numbers are listed in Appendix 1.

The combined dataset of nine plastid DNA regions was analysed using Bayesian Inference (BI) and Maximum Likelihood (ML). Three species of *Phlomis* were selected as outgroup, based on previous studies (Zhao et al. 2023a). The best-fit substitution model was selected by the jModelTest v.2.1.7 (Darriba et al. 2012) under the Akaike Information Criterion (AIC) score. BI and ML analyses were conducted on the Cyberinfrastructure for Phylogenetic Research Science (CIPRES) Gateway v.3.3 (Miller et al. 2010), using MrBayes (Ronquist et al. 2012) and RAxMLv.8.2.9 (Stamatakis 2014), respectively. Details for parameter settings follow the previous study of Zhao et al. (2021). All the phylogenetic trees with posterior probabilities (PP) and bootstrap values (BS) were exhibited and annotated in FigTree v.1.4.2 (Rambaut 2014).

### ﻿Morphological investigations

Species concept, definitions of characters and depiction generally follow Li and Hedge (1994). Type specimens and protologues for all species of *Phlomoides* in China were collated. Morphological features were based on herbarium as well as field investigations. Specimens at B, BM, C, CDBI, E, FI, GH, HIB, IBSC, K, KUN, LE, M, MA, MAO, MICH, MO, MW, NAS, P, PE, S, SG, TI, W, WUK and XJBI (herbarium acronyms followed Thiers 2022) and our collections from the field were examined for characterisation and morphological comparison. Additional morphological information (including habit, habitat, root, leaf, calyx, flower etc.) was taken from field observations, as well as literature (Hsuan 1977; Wu et al. 1977; Li 1985; Li and Hedge 1994).

Micro-features of leaf epidermis and floral bracts were investigated using Light Microscopy (LM) and Scanning Electron Microscopy (SEM). Photographs and morphological observations were taken using a Leica DM2500 light microscope (Leica Microsystems GmbH, Wetzlar, Germany). Mature leaves and floral bracts were taken from our collection (Table 1) for SEM investigation. Materials were mounted on to stubs and coated with gold, using a ZEISS EVO LS10 scanning electron microscope (Carl ZEISS NTS, Germany) with 10 kV voltage (Kunming Institute of Botany, Yunnan, China). Terminology of morphological characteristics of trichomes followed Khosroshahi and Salmaki (2019).

## ﻿Results

### ﻿Molecular phylogeny and systematic placement of *Phlomoideshenryi*

**Figure 1.**
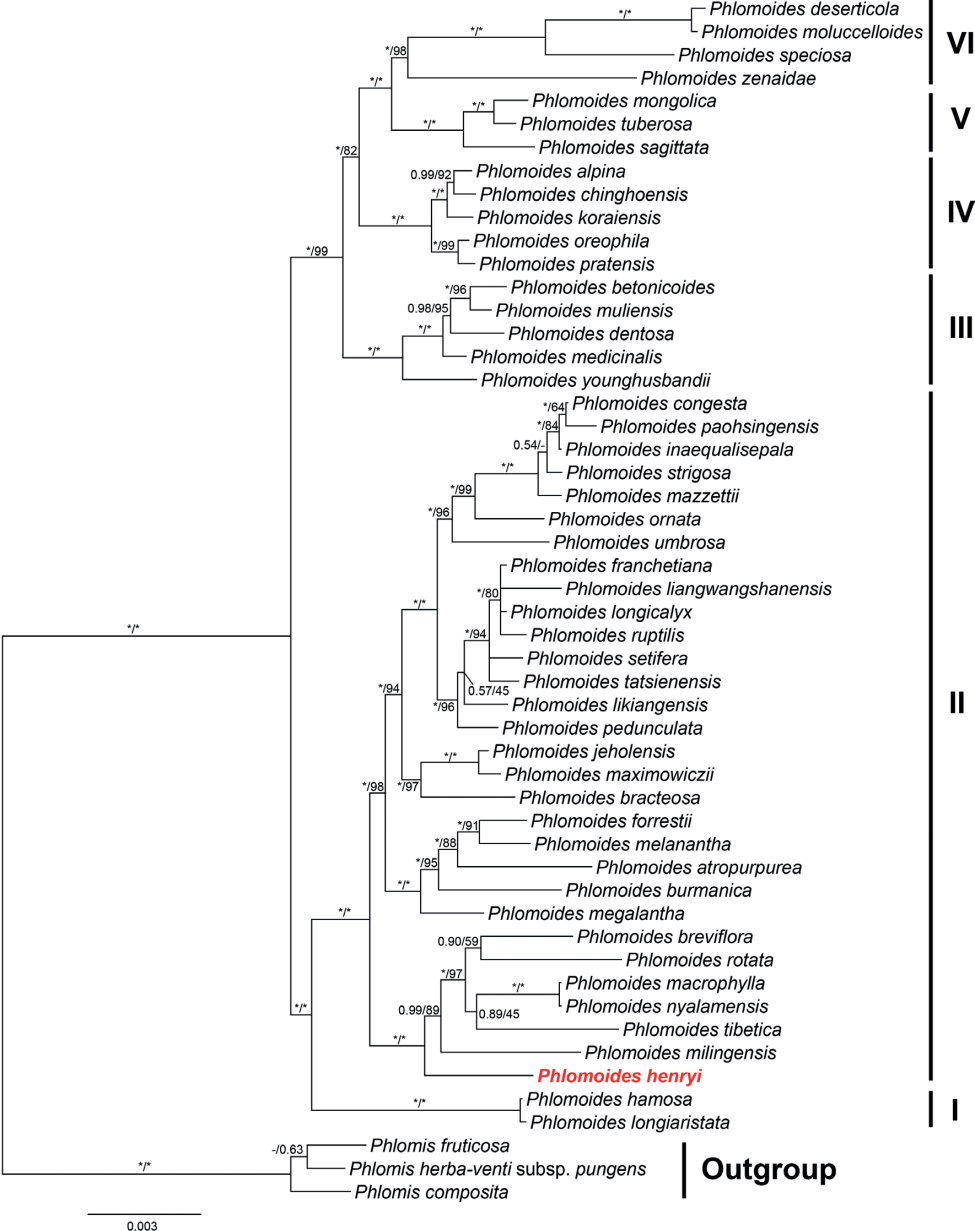
Phylogeny of *Phlomoides* inferred by Bayesian Inference (BI), based on the combined plastid dataset cpDNA. Support values displayed on the branches follow the order BI-PP/ML-BS (“ * ” indicates PP = 1.00 or BS = 100%, "-" indicates incongruent relationship between BI and ML tree.

A total of 18 sequences were newly sequenced in the present study and they were submitted to GenBank under accession nos. OR674852–OR674869. The aligned length of the combined plastid dataset was 9259 bp (2380 bp for *atpB*-*rbcL*, 421 bp for *psbA*-*trnH*, 1361 bp for *rpl16*, 681 bp for *rpl32-trnL*, 967 bp for *rps16*, 958 bp for *trnK*, 868 bp for *trnL-trnF*, 831 bp for *trnS-trnG* and 792 bp for *trnT-L*), respectively. The topologies of the BI and ML trees were consistent with each other, only the Bayesian 50% majority-rule consensus tree being presented, with the posterior probabilities (PP) and Bootstrap support (BS) and values being superimposed near the nodes (Fig. 1).

Monophyly of the genus *Phlomoides* was recovered (Fig. 1: PP =1.00/BS = 100%). The backbone topologies of *Phlomoides* recovered in present study are largely consistent with those of previous studies (Zhao et al. 2024), clade I is sister to Clade II with strong support values (Fig. 1: 1.00/100%), then sister to a large clade consisting of Clades III, IV, V and VI. Chinese *Phlomoides* species can subdivided into six clades (Fig. 1).

As shown in Fig. 1, the new species, *Phlomoideshenryi* is distantly related to *P.bracteosa*. Instead, *P.henryi* is sister to a subclade (Fig. 1: 1.00/100%) comprising *P.milingensis* (C.Y. Wu & H.W. Li) Kamelin & Makhm., *P.tibetica* (C. Marquand & Airy Shaw) Kamelin & Makhm., *P.macrophylla* (Wall.) Kamelin & Makhm., *P.nyalamensis* (H.W. Li) Y. Zhao & C.L. Xiang, *P.breviflora* (Benth.) Kamelin & Makhm., and *P.rotata* (Benth. ex Hook.f.) Mathiesen, while *P.bracteosa* is sister to *P.maximowiczii* (Regel) Kamelin & Makhm. and *P.jeholensis* (Nakai & Kitag.) Kamelin & Makhm.

### ﻿Trichome morphology of bracts and leaf epidermis

**Figure 2.**
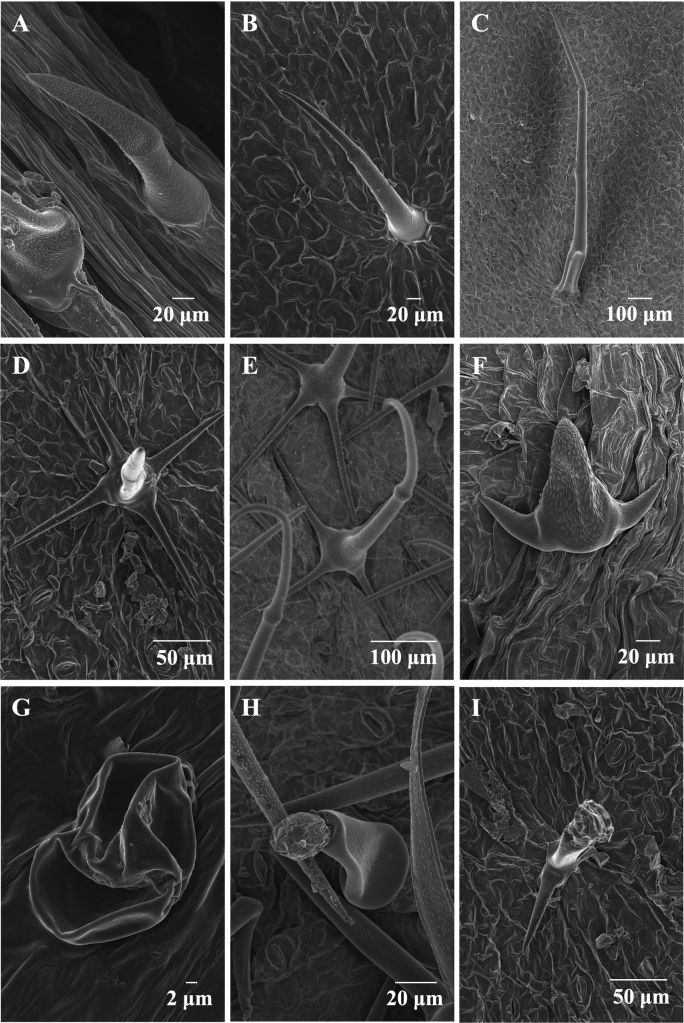
Different types of trichomes of *Phlomoides***A** short simple non-glandular trichomes (*P.macrophylla*) **B** short simple non-glandular trichomes (*P.breviflora*) **C** long simple non-glandular trichomes (*P.henryi*) **D** symmetrically non-glandular stellate (*P.breviflora*) **E** non-glandular stellate with central long branch (*P.bracteosa*) **F** bi- or trifurcate non-glandular stellate (*P.nyalamensis*) **G** sub-sessile/ sessile glandular trichomes (*P.macrophylla*) **H** simple glandular trichomes of (*P.bracteosa*) **I** branched glandular trichomes (*P.breviflora*).

Two basic types of trichomes can be observed in *Phlomoides*: eglandular hairs and glandular hairs, as reported by Khosroshahi and Salmaki (2019). Both kinds of hairs can be divided into simple (unbranched) and branched trichomes. Simple eglandular trichomes were subdivided by length: short (< 500 μm) (Fig. 2A, B), long (500–2000 μm) (Fig. 2C) and extremely long (> 2000 μm); branched eglandular trichomes were subdivided into symmetrically stellate (Fig. 2D), stellate with central long branch (Fig. 2E) and bi- or trifurcate (Fig. 2F). Simple glandular trichomes were subdivided by stalk length: sub-sessile/sessile glandular trichomes (Fig. 2G), short-stalked glandular trichomes (< 500 μm) (Fig. 2H) and long-stalked glandular trichomes (> 500 μm). Branched glandular trichomes contain only one type (Fig. 2I). For the eight species examined in this study, extremely long simple non-glandular trichomes and long-stalked glandular trichomes were not observed.

**Figure 3.**
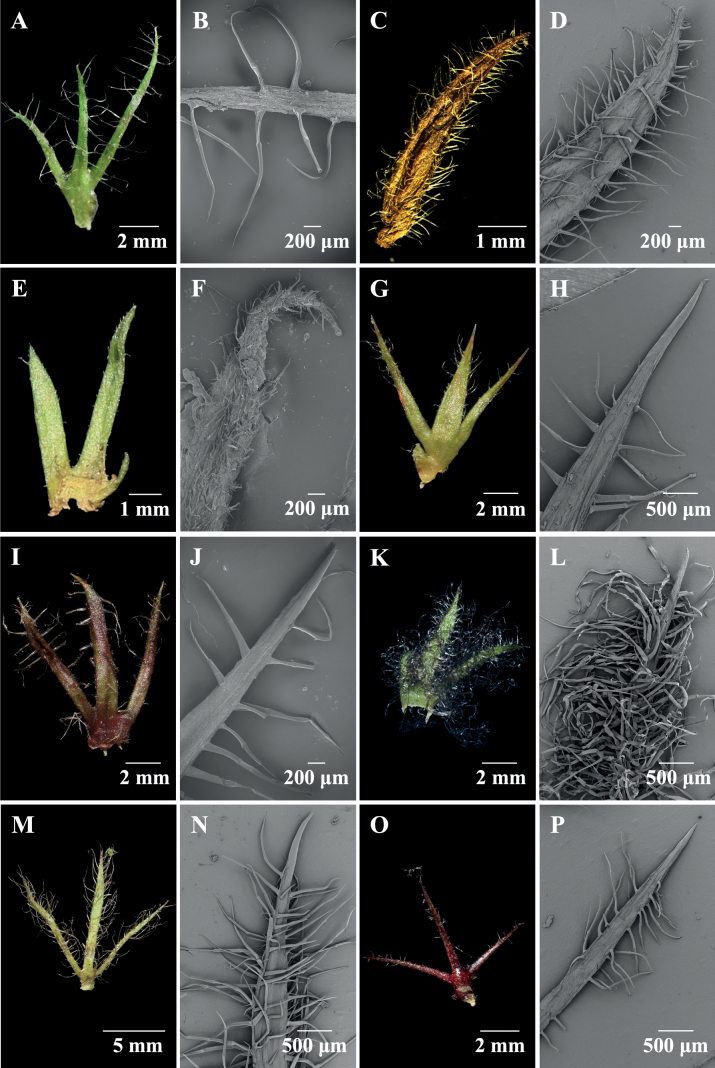
Photos of bracts, SEM of bracts of *Phlomoideshenryi* and related species **A, B***P.henryi***C, D***P.bracteosa***E, F***P.breviflora***G, H***P.macrophylla***I, J***P.nyalamensis***K, L***P.tibetica***M, N***P.milingensis***O, P***P.rotata*. **A, C, E, G, I, K, M, O** photos of bracts **B, D, F, H, J, L, N, P**SEM of bracts.

**Figure 4.**
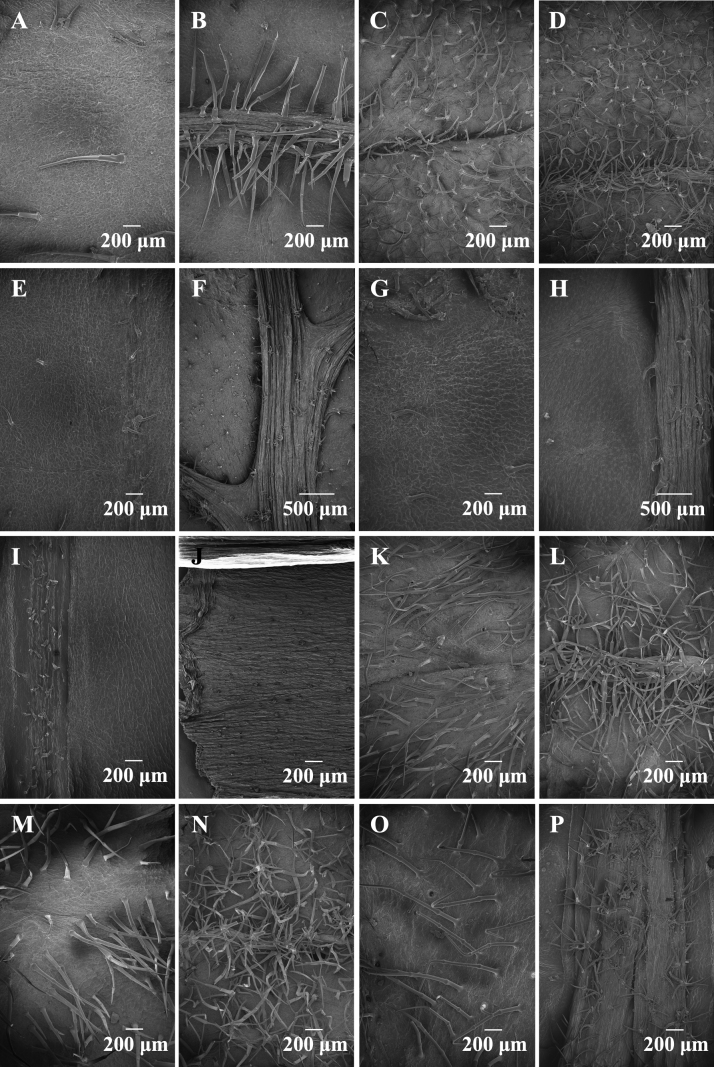
SEM of both sides of leaves of *Phlomoideshenryi* and related species **A, B***P.henryi***C, D***P.bracteosa***E, F***P.breviflora***G, H***P.macrophylla***I, J***P.nyalamensis***K, L***P.tibetica***M, N***P.milingensis***O, P***P.rotata***A, C, E, G, I, K, M, O**SEM of adaxial leaves **B, D, F, H, J, L, N, P**SEM of abaxial leaves.

Figs 3, 4 and Table 2 show the morphology and distribution of trichomes on leaves and bracts of the investigated taxa. Sub-sessile/sessile glandular trichomes occur widely in every part of each species of *Phlomoides* (Table 2). Short stalked glandular trichomes were observed on the abaxial leaf surface in five species and on the bracts of only one species, i.e. *P.breviflora*. Branched glandular trichomes were only recorded on the abaxial leaf surface of *P.breviflora*.

**Table 2.** Distribution of different types of trichome in the examined *Phlomoides* spp.

Tissue	Species	Eglandular hairs					Glandular hairs		
		Simple		Branched			Simple		Branched
		Short	Long	Symmetrically stellate	Stellate with central long branch	Bi- or trifurcate	Sub-sessile/ sessile	Short stalked	
**Adaxial leaf**	*P.henryi*	+	+	–	–	–	+	–	–
	*P.bracteosa*	+	–	+	+	+	+	–	–
	*P.breviflora*	+	–	–	–	–	+	–	–
	*P.macrophylla*	+	–	–	–	–	+	–	–
	*P.nyalamensis*	+	–	–	–	–	+	–	–
	*P.tibetica*	+	+	–	–	–	+	–	–
	*P.milingensis*	+	+	–	–	–	+	–	–
	*P.rotata*	+	+	–	–	–	+	–	–
**Abaxial leaf**	*P.henryi*	+	+	–	–	–	+	–	–
	*P.bracteosa*	+	–	+	+	+	+	+	–
	*P.breviflora*	+	–	+	+	+	+	+	+
	*P.macrophylla*	+	–	+	+	+	+	+	–
	*P.nyalamensis*	–	–	–	–	+	+	–	–
	*P.tibetica*	+	+	–	+	–	+	+	–
	*P.milingensis*	+	–	–	+	+	+	+	–
	*P.rotata*	+	–	+	+	+	+	–	–
**Bract**	*P.henryi*	+	+	–	–	–	+	–	–
	*P.bracteosa*	+	+	+	+	+	+	–	–
	*P.breviflora*	+	–	+	+	–	+	+	–
	*P.macrophylla*	+	+	–	–	–	+	–	–
	*P.nyalamensis*	+	+	–	–	–	+	–	–
	*P.tibetica*	+	+	–	–	–	+	–	–
	*P.milingensis*	+	+	–	+	+	+	–	–
	*P.rotata*	+	+	–	–	–	+	–	–

Simple short eglandular trichomes were observed in every species on leaf and bract surface, but were missing in the abaxial leaf of *Phlomoidesnyalamensis*, since it was nearly glabrous (Fig. 4J). Adaxial leaf surfaces were often covered by simple eglandular trichomes, except for *P.bracteosa* (Fig. 4C), which has dense branched eglandular trichomes on the adaxial leaf surface. Simple long eglandular trichomes were most common on bracts (Fig. 3B, H, J, L, O). Abaxial leaf surfaces often had branched eglandular trichomes, but these are not present in the new species (Fig. 4B).

Trichomes were transparent to white or brown to black in *Phlomoides*. Trichomes inside the upper corolla lip of the new species (*P.henryi*), *P.bracteosa* and *P.rotata* were transparent to white, while the other five species were brown to black. Bract trichomes of *P.tibetica* and *P.milingensis* were brown to black (Fig. 3K, M), the other six species were transparent to white (Fig. 3A, C, E, G, I, O).

## ﻿Discussion

Herbaria house millions of specimens that embody the plant diversity on the Earth. Many new species have been lurking in herbaria for many years before being published. Bebber et al. (2010) estimated that 84% of new species’ descriptions were from old specimens collected more than five years prior to publication and 25% from specimens more than 50 years old. During the taxonomic review of some groups of Lamiaceae, we have also found some new species from old herbarium specimens (Chen et al. 2014; Dong et al. 2015), indicating taxonomic work, based on herbaria, is still a very important resource for the discovery of new taxa.

The resulting phylogenetic tree of *Phlomoides* in this study was similar to that in previous study (Zhao et al. 2024). The new species, *P.henryi*, was nested within Clade II and formed a separate branch (Fig. 1: 1.00/100%) that is sister to a subclade containing *P.rotata* and five species with brown to black trichomes on the upper corolla. Geographically, *Phlomoideshenryi* is distributed in southern Yunnan, while the other six species in this subclade were mainly distributed in the Qinghai-Tibetan Plateau and Himalaya. The new species is morphologically distinct from the other six species in this subclade. For example, trichomes on the upper corolla lip of *P.henryi* and *P.rotata* are colourless and perceptually transparent to white, but brown to black in the other five species. Morphologically, *P.rotata* is distinct from all other species of *Phlomoides* by the very short stem producing a rosette of leaves with the plant often less than 10 cm high, while *P.henryi* is generally taller than 1 m. As we observed, all the species with trichomes brown to black were embedded within this subclade. The sister clade to that containing *P.henryi* contains 23 species that are mainly distributed in Hengduan Mountains. *Phlomoideshenryi* is similar to other species in Clade I and Clade II in lacking basal leaves. Only four species have basal leaves in Clade II, i.e. *P.rotata*, *P.tibetica*, *P.milingensis* and *P.atropurpurea*, while all the species in Clades III–VI have basal leaves.

As above mentioned, we believe that the differences merit recognition of the new species and we describe it below.

**Figure 5.**
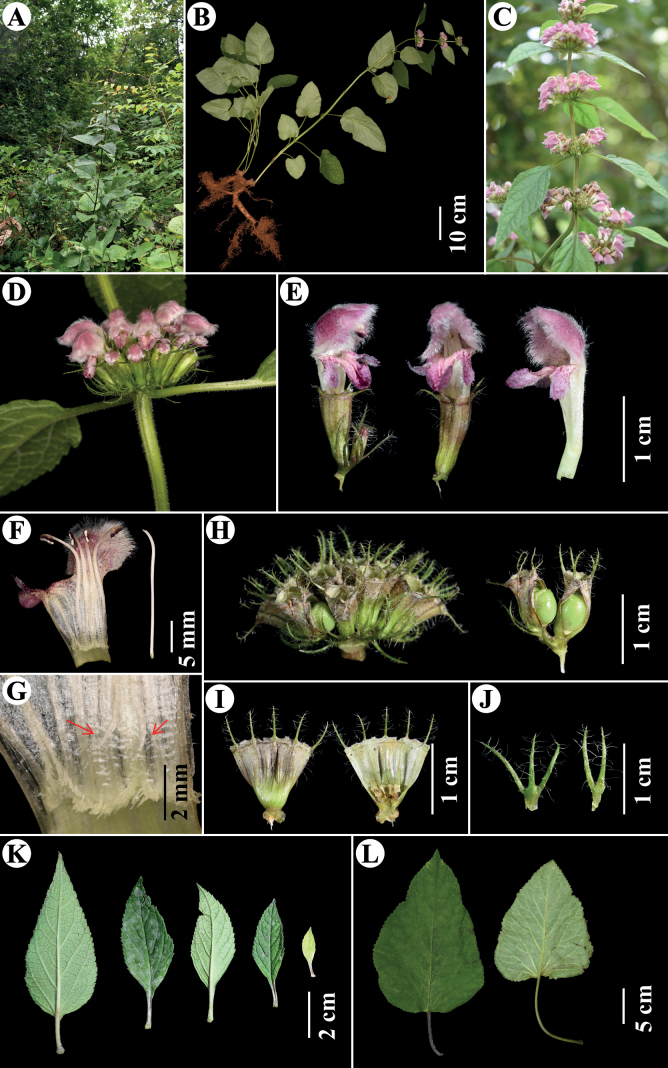
*Phlomoideshenryi* Y.Zhao & C.L.Xiang **A** habitat **B** plant with linear-tuberous roots **C** inflorescence **D** verticillaster **E** flowers **F** dissected flower **G** appendages at base of posterior filaments **H** fruiting calyces **I** dissected calyces **J** bracts **K** floral leaves **L** stem leaves. Photographs by Yue Zhao, except C by Li Chen.

*Phlomoides* is a morphologically diverse and taxonomically difficult group with many characters used for traditional taxonomy being highly variable. In this study, we investigated trichome micro-morphology on bracts and leaves of *Phlomoideshenryi* and related species. We found that trichomes are a useful character to distinguish some morphologically similar species. Based on the colour of trichomes, we can separate two groups of those species. *Phlomoidesnyalamensis*, *P.macrophylla*, *P.tibetica*, *P.milingensis* and *P.breviflora* have brown to black trichomes on the upper corolla lip, while the other species (*P.rotata*, *P.bracteosa* and the new species described here, *P.henryi*) have transparent to white trichomes on the upper corolla lip. Trichome density and bract trichome colour can separate *P.tibetica* from the similar *P.milingensis*. Both species are distributed in Xizang at an altitude from 3500–4500 m and Hsuan (1977) placed them within Series *Tibeticae*. *Phlomoidestibetica* has floral bracts with black simple trichomes and no branched trichomes, while *P.milingensis* has floral bracts with brown simple and branched trichomes. Similarly, the new species described here, *P.henryi*, can be distinguished from the six related species in the subclade by the absence of branched trichomes on the abaxial leaf surface (Fig. 5B). *Phlomoidesbracteosa* can easily be separated from these six species by having branched trichomes on the adaxial leaves (Fig. 5C). Azizian and Cutler (1982) have found that adaxial and abaxial leaf surfaces have different trichome types, but in that work, *Phlomoides* was treated as a section of *Phlomis* and they only discussed the differences amongst Phlomissect.Phlomis, Phlomissect.Phlomoides and *Eremostachys* and not at the species level. Subsequent studies did not observe trichomes on different structures (Seyedi and Salmaki 2015; Khosroshahi and Salmaki 2019). However, here we found different structures were covered with significantly different trichomes and these differences can be used as evidence to separate morphologically similar species. Future studies should focus on micro-morphological investigation of trichomes and other characters (i.e. appendages, calyces, roots, mericarps) and those micro-features are probably helpful for taxonomy and species identification of *Phlomoides* species.

In order to distinguish those species grouped with the new species in the phylogenetic tree (Fig. 2), as well as *P.bracteosa*, we provide a key, mainly based on macro- and micro-morphological trichomes.

### ﻿Key to *P.henryi* and morphologically similar species

**Table:** 

1	Upper corolla lip with trichome transparent to white	**2**
–	Upper corolla lip with trichomes brown to black	**4**
2	No branched trichomes on leaf and bract	***P.henryi***
–	With branched trichomes on leaf and bract	**3**
3	Floral bracts with branched trichomes, bracts linear to lanceolate	***P.bracteosa***
–	Floral bracts with simple trichomes only, bracts needle-like to subulate	***P.rotata***
4	Floral bracts with trichomes brown to black	**5**
–	Floral bracts with trichomes transparent to white	**6**
5	Floral bracts with black simple trichomes, no branched trichomes	***P.tibetica***
–	Floral bracts with brown simple and branched trichomes	***P.milingensis***
6	Floral bracts less than 1 cm long, with branched trichomes	***P.breviflora***
–	Floral bracts more than 1 cm long, with only simple trichomes	**7**
7	Flower purple	***P.nyalamensis***
–	Flower white	***P.macrophylla***

### ﻿Taxonomic treatment

#### ﻿ Phlomoides henryi

Taxon classificationPlantaeLamialesLamiaceae

Y.Zhao & C.L.Xiang sp. nov.

952A4EFD-8DF8-5080-AC6E-666D6472F87A

urn:lsid:ipni.org:names:77330835-1

Fig. 5

##### Type.

China, Yunnan Province, Honghe Hani and Yi Autonomous Prefecture, Jianshui County, on the forest edge, 23°57′52.54″N, 102°59′49.47″E, alt. 1279 m, 3 Sep 2022, *F. Zhao, Y. Zhao & C.L. Xiang XCL2222* (holotype: KUN!; isotypes: KUN!, CSH!).

##### Diagnosis.

Within the subclade, *Phlomoideshenryi* is morphologically similar to *P.rotata* for having transparent to white trichomes inside the upper corolla lip rather than brown to black and is distinct from all other species by lacking branched hairs. *P.bracteosa* has similar transparent to white trichomes inside the upper corolla lip, but with branched trichomes on both sides of leaves and floral bracts. The differences between *P.henryi*, *P.rotata* and *P.bracteosa* are listed in Table 3.

**Table 3.** Morphological comparisons amongst *Phlomoideshenryi*, *P.rotata* and *P.bracteosa*.

Characters	*P.henryi*	*P.rotata*	*P.bracteosa*
**Height**	100–150 cm	2.5–10 cm	20–50 (–100) cm
**Basal leaves**	absent	rosette basal leaves	absent
**Floral leaf shape**	narrowly lanceolate	lanceolate, oblanceolate, or linear	Ovate to lanceolate
**Floral leaf petiole length**	5–35 mm	Lack obvious petiole	Upper floral leaves sessile, lower floral leaves with petiole 5–20 mm long
**Floral bracts shape**	Subulate	Needle-like to subulate	Linear to lanceolate, often with enlarged bracts
**Branched trichomes**	No branched trichomes	With branched trichomes on abaxial leaves	With branched trichomes on both sides of leaves and bracts

##### Perennial herbs.

Roots robust, linear-tuberous. Stems 1–1.5 m tall, subquadrangular, robust, simple pilose. Basal leaves absent; stem leaves with petioles 4–15 cm long, with simple trichomes, broadly ovate to ovate-oblong, papery, 10–18 × 15–24 cm, adaxially green with sparse simple trichomes, abaxially light green, with sparse simple trichomes, denser and longer on the main vein, base cordate, margin serrate or crenate, apex acute to acuminate. Verticillasters axillary, 8–20-flowered; floral leaves with petioles 5–35 mm long, lanceolate, base rounded to cuneate, 1–6 × 0.5–4 cm, gradually reduced upwards; bracts subulate, 6–10 mm long, with sparse long simple trichomes, ca. 2 mm long. Calyx tubular, 10–11 × 4–5 mm, pubescent outside with sparse long simple trichomes on veins, conspicuously 10-veined; teeth 5, truncate, ca. 1.5 mm long, apical spines 3–4 mm long, with sparse long simple trichomes. Corolla light purple to pink, ca. 2.1 cm long, 2-lipped; posterior lip ca. 7 mm long, galeate, densely stellate tomentose outside, margin denticulate, bearded inside; anterior lip 3-lobed, ca. 7 × 8 mm, middle lobe largest, oblong, ca. 5 × 3 mm, lateral lobes ovate; tube glabrous outside, ca. 1.5 cm, annulate pilose inside. Stamens 4, included, with cobwebby indumentum, posterior filaments with reflexed appendages at base. Style unequally 2-lobed. Nutlets oblong-globose, glabrous.

##### Etymology.

The new species is named after the collector Augustine Henry (1857–1930), who collected more than 15,000 dry specimens and seeds from China.

##### Phenology.

Flowering from July to September and fruiting from October to December.

##### Distribution, habitat and ecology.

Based on present collections, *P.henryi* is only known from its type locality, i.e. Muyang Mountain in Jianshui County, Yunnan Province, China. It is restricted to the edge of the forest at an elevation near 1280 m.

##### Chinese name.

jiàn shuǐ cǎo cāo sū (建水草糙苏).

##### Additional specimen examined.

*Phlomoideshenryi*: ***Paratypes*.** China, Yunnan Province, Honghe Hani and Yi Autonomous Prefecture, Jianshui County, 6 September 2019, *Jianshui Exped. 2164* (KUN); Yunnan Province, Mengtze, 1898, *A. Henry 10216* (K000928267, K without barcode, LE without barcode); Yunnan Province, “Lao Kouy [Chan], 11 August 1907, *F. Ducloux 369* (E).

*Phlomoidesbracteosa*: India. Choor & Kidarlonta, 1832, *J.F. Royle 633* (Type: K, K000894384); State of Punjab: Kangra, Lahul, Bhaga Valley, alt. 3000 m, 26 July 1933, *T.R. Chand 74A* (MICH, 1519061); State of Uttarakhand: Bhyundar Valley – Valley of flowers (N.E. road Josimath to Badrinath), alt. 3430 m, 16 August 1975, *Anonymous 6583* (PE, 1290791).

*Phlomoidesbreviflora*: China. Xizang Province: Yadong County, on the way from Yadong to Dingga, alt. 2850 m, 11 June 1975, *Qinghai-Tibet Exped. 750283* (Holotype: KUN, 1218974!; Isotype: PE, 00835569!, 00835570!); Nielamu County, Qu Town, alt. 3240 m, 12 September 2019, *Y.P. Chen, Y. Zhao & B.Y. Zhang EM1139* (KUN). India. Sikkim: Nayathang-Phalut, alt. 3500 m, 2 June 1960, *H. Hara et al. 5551* (TI); West Bengal: Darjeeling, Phalut, alt. 3500 m, 5 June 1960, *H. Hara et al. 5555* (TI); Darjeeling, Garibans-Tanglu, alt. 2700 m, 7 June 1969, *H. Hara et al. 5557* (TI). Nepal. Bagmati Province: Rasuwa District, Gossain than (Gosainkund), Benth in Wall. *Cat. Herb. Ind. n. 2066* (Type: K, K001115039!).

*Phlomoidesmacrophylla*: Nepal. Gandaki Province: Mustang, Annapurna Conservation area, Trekking route Jomosom-Nayapul, Near Ghorepani village (way to Tikhedhunga). China. Xizang Province: Yadong County, Shang Yadong Village, alt. 3448 m, 30 August 2023, *Y. Zhao et al. XCL2703* (KUN); Yadong County, on the way from Yadong County to the Pass of Naiduila Mountain, 9 September 2019, *Y.P. Chen, Y. Zhao & B.Y. Zhang EM1145* (KUN).

*Phlomoidesnyalamensis*: China. Xizang Province: Nyalam County, Zhangmu Town, on the way from Lixin to Xuebugang, open spaces in forests, alt. 2700–2800 m, 29 June 1975, *Qinghai-Xizang Comp. Exped. 6622* (Holotype, KUN 1218985! Isotype, KUN 1218984!); Nyalam County, Zhangmu Town, Lixin Village, damp hillside, alt. 2800 m, 24 August 1972, *Tibetan herbal medicine Exped. 1461* (PE, 00835826); Nyalam County, Zhangmu Town, on the way from Lixin to Xuebugang, edge of forests, alt. 2896 m, 13 September 2019, *Y.P. Chen, Y. Zhao & B.Y. Zhang EM1145* (KUN).

*Phlomoidestibetica*: China. Xizang Province: in open alpine pastures of turf in well drained situations, alt. 4200–4500 m, 6 July 1924, *F. K. Ward 5901* (Holotype: K, K000894378!; isotype: BM, BM000950520, E, E00301982!); Linzhi City, Bomi County, Northern Galongla Mountain, 3800–3900 m, 16 August 1982, *S.Z. Cheng & B.S. Li 00160* (PE, 00923472); Linzhi City, on the way from Lulang Town to the Pass of Sejila Mountain, 4108 m, 15 September 2016, *C.L. Xiang et al. 1456* (KUN). Bhutan. Trashigang District, Shingbe Town, 3800 m, 27 May 1949, *F. Ludlow, G. Sherriff & J.H. Hicks 20673* (BM).

*Phlomoidesmilingensis*: China. Xizang Province: Mainling County, Zedanggang, alt. 4400 m, 26 July 1972, *Tibetan herbal medicine Exped. 3883* (holotype: PE, 00835583; isotype: PE, 00923457); Mainling County, Nanyi Mountain, alt. 3400–3500 m, 28 July 1972, *Tibetan herbal medicine Exped. 4228* (PE, 00835584; PE, 00923459); Gongbo’gyamda County, Xueka, 10 August 1974, *Anonymous 2129* (PE, 00832244).

*Phlomoidesrotata*: China: Xizang, Shannan City, Cona County, near radar station, alt. 4280 m, 17 July 1975, *C.Y. Wu et al. 75-893* (KUN, 0216402); Naqu City, Lhari County, meteorological station, alt. 4500 m, 8 June 1976, *Qinghai-Xizang Comp. Exped. 10491* (KUN, 0216409); Nyingchi City, Gongbo’gyamda County, Mira pass, alt. 4920 m, 30 August 1974, *Qinghai-Xizang Comp. Exped. 74-2027* (KUN, 0216384); Sichuan, Liangshan Yi Autonomous Prefecture, Muli County, on the way from Mogalaji to Nonsa pasture, alt. 4312 m, 30 August 2015, *C.L. Xiang 1219* (KUN, 1264607); Yunnan, Diqing Tibetan Autonomous Prefecture, Deqin County, Baimaxueshan, alt. 4300 m, 4 June 2000, *Z.K. Zhou et al. 159* (KUN, 0699320); Qinghai, Yushu Tibetan Autonomous Prefecture, Yushu County, Hongtu Mountain, alt. 4900 m, 3 September 2013, *J.W. Zhang et al. Zh678* (KUN, 1260768).

## Supplementary Material

XML Treatment for Phlomoides henryi

## ﻿Additional information

### Author ORCIDs

Yue Zhao https://orcid.org/0000-0001-6850-5070

Fei Zhao https://orcid.org/0000-0001-7328-2262

Alan J. Paton http://orcid.org/0000-0002-6052-6675

Jin-Fei Xiao https://orcid.org/0000-0001-7729-2548

Ya-Ping Chen http://orcid.org/0000-0002-7502-1848

Chun-Lei Xiang https://orcid.org/0000-0001-8775-6967

### Data availability

DNA sequences associated with this work are available from GenBank (https://www.ncbi.nlm.nih.gov/genbank/) or from the corresponding author upon reasonable request.

## ﻿Appendix 1

Specimen information (taxon, voucher, herbarium, country) for samples newly-sequenced in the present study with GenBank accession numbers for *atpB-rbcL*, *psbA-trnH*, *rpl16*, *rpl32-trnL*, *rps16*, *trnK*, *trnL-trnF*, *trnS-trnG* and *trnT-L*, respectively. Herbarium abbreviations are listed after the vouchers. The accession numbers marked with an asterisk represent sequences newly generated. Only GenBank accession numbers are listed for sequences downloaded from NCBI.

***Phlomiscomposita*** Pau, ON820555, ON820616, ON835580, ON835627, ON835674, ON835721, ON843184, OR632134, ON843231; ***Phlomisfruticosa*** L., ON820556, ON820615, ON835581, ON835628, ON835675, ON835722, ON843185, OR632135, ON843232; ***Phlomisherba-venti*** subsp. ***pungens*** (Willd.) Maire ex DeFilipps, ON820557, ON820614, ON835582, ON835629, ON835676, ON835723, ON843186, ON815620, ON843233; ***Phlomoidesalpina*** (Pall.) Adylov, Kamelin & Makhm., OR631925, OR631967, OR642305, OR632009, OR632051, OR642347, OR642389, OR632137, OR632093; ***Phlomoidesatropurpurea*** (Dunn) Kamelin & Makhm., OQ672946, OQ672946, OQ672946, OQ672946, OQ672946, OQ672946, OQ672946, OQ672946, OQ672946; ***Phlomoidesbetonicoides*** (Diels) Kamelin & Makhm., MN617020, MN617020, MN617020, MN617020, MN617020, MN617020, MN617020, MN617020, MN617020; ***Phlomoidesbracteosa*** (Royle ex Benth.) Kamelin & Makhm., *C.R. Lancaster 72* (BM), India, Srinagar, Pahalgam, Gully, alt. 2286 m, OR674852*, OR674855*, OR674856*, OR674858*, OR674860*, OR674863*, OR674864*, OR674867*, OR674869*; ***Phlomoidesbreviflora*** (Benth.) Kamelin & Makhm., OQ672923, OQ672923, OQ672923, OQ672923, OQ672923, OQ672923, OQ672923, OQ672923, OQ672923; ***Phlomoidesburmanica*** (Mukerjee) Kamelin & Makhm., ON820563, ON820630, ON835588, ON835635, ON835682, ON835729, ON843192, OR632142, ON843239; ***Phlomoideschinghoensis*** (C.Y. Wu) Kamelin & Makhm., ON820580, ON820611, ON835605, ON835652, ON835699, ON835746, ON843209, ON815622, ON843256; ***Phlomoidescongesta*** (C.Y. Wu) Kamelin & Makhm., ON820567, ON820608, ON835592, ON835639, ON835686, ON835733, ON843196, OR632145, ON843243; ***Phlomoidesdentosa*** (Franch.) Kamelin & Makhm., OR631929, OR631973, OR642309, OR632015, OR632057, OR642351, OR642395, OR632149, OR632099; ***Phlomoidesdeserticola*** Sennikov, OQ672935, OQ672935, OQ672935, OQ672935, OQ672935, OQ672935, OQ672935, OQ672935, OQ672935; ***Phlomoidesforrestii*** (Diels) Kamelin & Makhm., OR631934, OR631976, OR642313, OR632018, OR632060, OR642355, OR642398, OR632153, OR632102; ***Phlomoidesfranchetiana*** (Diels) Kamelin & Makhm., ON820561, ON820621, ON835586, ON835633, ON835680, ON835727, ON843190, OR632155, ON843237; ***Phlomoideshamosa*** (Benth.) Mathiesen, ON820558, ON820604, ON835583, ON835630, ON835677, ON835724, ON843187, OQ672937, ON843234; ***Phlomoideshenryi*** Y.Zhao & C.L.Xiang, *F. Zhao, Y. Zhao & C.L. Xiang XCL2222* (KUN), China, Yunnan Province, Jianshui County, Limin Town, Muyang Mountain, alt. 2179 m, OR674853*, OR674854*, OR674857*, OR674859*, OR674861*, OR674862*, OR674865*, OR674866*, OR674868*; ***Phlomoidesinaequalisepala*** (C.Y. Wu) Kamelin & Makhm., OR631937, OR631979, OR642317, OR632021, OR632063, OR642359, OR642401, OR632160, OR632105; ***Phlomoidesjeholensis*** (Nakai & Kitag.) Kamelin & Makhm., OR631938, OR631980, OR642318, OR632022, OR632064, OR642360, OR642402, OR632162, OR632106; ***Phlomoideskoraiensis*** (Nakai) Kamelin & Makhm., OR631939, OR631981, OR642319, OR632023, OR632065, OR642361, OR642403, OR632163, OR632107; ***Phlomoidesliangwangshanensis*** Y. Zhao, H.L. Zheng & C.L. Xiang, OR631940, OR631982, OR642320, OR632024, OR632066, OR642362, OR642404, OR632165, OR632108; ***Phlomoideslikiangensis*** (C.Y. Wu) Kamelin & Makhm., OR631942, OR631984, OR642322, OR632026, OR632068, OR642364, OR642406, OR632167, OR632110; ***Phlomoideslongiaristata*** (C.Y. Wu & H.W. Li) Salmaki, ON820559, ON820603, ON835584, ON835631, ON835678, ON835725, ON843188, ON815625, ON843235; ***Phlomoideslongicalyx*** (C.Y. Wu) Kamelin & Makhm., OR631943, OR631985, OR642323, OR632027, OR632069, OR642365, OR642407, OR632168, OR632111; ***Phlomoidesmacrophylla*** (Benth.) Kamelin & Makhm., OR631944, OR631986, OR642324, OR632028, OR632070, OR642366, OR642408, OR632169, OR632112; ***Phlomoidesmaximowiczii*** (Regel) Kamelin & Makhm., ON820565, ON820622, ON835590, ON835637, ON835684, ON835731, ON843194, OR632170, ON843241; ***Phlomoidesmazzettii*** Lazkov, OR631945, OR631987, OR642325, OR632029, OR632071, OR642367, OR642409, OR632171, OR632113; ***Phlomoidesmedicinalis*** (Diels) Kamelin & Makhm., OR631946, OR631988, OR642326, OR632030, OR632072, OR642368, OR642410, OR632172, OR632114; ***Phlomoidesmegalantha*** (Diels) Kamelin & Makhm., OR631947, OR631989, OR642327, OR632031, OR632073, OR642369, OR642411, OR632173, OR632115; ***Phlomoidesmelanantha*** (Diels) Kamelin & Makhm., OR631948, OR631990, OR642328, OR632032, OR632074, OR642370, OR642412, OR632174, OR632116; ***Phlomoidesmilingensis*** (C.Y. Wu & H.W. Li) Kamelin & Makhm., OR631949, OR631991, OR642329, OR632033, OR632075, OR642371, OR642413, OR632175, OR632117; ***Phlomoidesmoluccelloides*** (Bunge) Salmaki, OQ672938, OQ672938, OQ672938, OQ672938, OQ672938, OQ672938, OQ672938, OQ672938, OQ672938; ***Phlomoidesmongolica*** (Turcz.) Kamelin & A.L. Budantzev, ON820576, ON820617, ON835601, ON835648, ON835695, ON835742, ON843205, OR632176, ON843252; ***Phlomoidesmuliensis*** (C.Y. Wu) Kamelin & Makhm., OR631950, OR631992, OR642330, OR632034, OR632076, OR642372, OR642414, OR632177, OR632118; ***Phlomoidesnyalamensis*** (H.W. Li) Y. Zhao & C.L. Xiang, OR631952, OR631994, OR642332, OR632036, OR632078, OR642374, OR642416, OR632179, OR632120; ***Phlomoidesoreophila*** (Kar. & Kir.) Adylov, Kamelin & Makhm., OR631953, OR631995, OR642333, OR632037, OR632079, OR642375, OR642417, OR632180, OR632121; ***Phlomoidesornata*** (C.Y. Wu) Kamelin & Makhm., ON820570, ON820618, ON835595, ON835642, ON835689, ON835736, ON843199, OR632181, ON843246; ***Phlomoidespaohsingensis*** (C.Y. Wu) Kamelin & Makhm., OR631954, OR631996, OR642334, OR632038, OR632080, OR642376, OR642418, OR632182, OR632122; ***Phlomoidespedunculata*** (Y.Z.Sun) Kamelin & Makhm., OQ672936, OQ672936, OQ672936, OQ672936, OQ672936, OQ672936, OQ672936, OQ672936, OQ672936; ***Phlomoidespratensis*** (Kar. & Kir.) Adylov, Kamelin & Makhm., ON820579, ON820612, ON835604, ON835651, ON835698, ON835745, ON843208, ON815626, ON843255; ***Phlomoidesrotata*** (Benth. ex Hook.f.) Mathiesen, ON820564, ON820602, ON835589, ON835636, ON835683, ON835730, ON843193, ON815627, ON843240; ***Phlomoidesruptilis*** (C.Y. Wu) Kamelin & Makhm., OR631955, OR631997, OR642335, OR632039, OR632081, OR642377, OR642419, OR632183, OR632123; ***Phlomoidessagittata*** (Regel) C.L.Xiang & Y.Zhao, ON820578, ON820620, ON835603, ON835650, ON835697, ON835744, ON843207, ON815617, ON843254; ***Phlomoidessetifera*** (Bureau & Franch.) Kamelin & Makhm., OR631956, OR631998, OR642336, OR632040, OR632082, OR642378, OR642420, OR632186, OR632124; ***Phlomoidesspeciosa*** (Rupr.) Adylov, Kamelin & Makhm., ON820592, ON820631, ON835617, ON835664, ON835711, ON835758, ON843221, ON815629, ON843268; ***Phlomoidesstrigosa*** (C.Y. Wu) Kamelin & Makhm., OR631957, OR631999, OR642337, OR632041, OR632083, OR642379, OR642421, OR632187, OR632125; ***Phlomoidestatsienensis*** (Bureau & Franch.) Kamelin & Makhm., OR631960, OR632002, OR642340, OR632044, OR632086, OR642382, OR642424, OR632190, OR632128; ***Phlomoidestibetica*** (C. Marquand & Airy Shaw) Kamelin & Makhm., OR631961, OR632003, OR642341, OR632045, OR632087, OR642383, OR642425, OR632193, OR632129; ***Phlomoidestuberosa*** (L.) Moench, ON820575, ON820624, ON835600, ON835647, ON835694, ON835741, ON843204, ON815631, ON843251; ***Phlomoidesumbrosa*** (Turcz.) Kamelin & Makhm., ON820571, ON820605, ON835596, ON835643, ON835690, ON835737, ON843200, OR632198, ON843247; ***Phlomoidesyounghusbandii*** (Mukerjee) Kamelin & Makhm., MW405448, MW405448, MW405448, MW405448, MW405448, MW405448, MW405448, MW405448, MW405448; ***Phlomoideszenaidae*** (Popov) Adylov, Kamelin & Makhm., OQ672945, OQ672945, OQ672945, OQ672945, OQ672945, OQ672945, OQ672945, OQ672945, OQ672945.
